# Network Mapping of Connectivity Alterations in Disorder of Consciousness: Towards Targeted Neuromodulation

**DOI:** 10.3390/jcm9030828

**Published:** 2020-03-18

**Authors:** Lucia Mencarelli, Maria Chiara Biagi, Ricardo Salvador, Sara Romanella, Giulio Ruffini, Simone Rossi, Emiliano Santarnecchi

**Affiliations:** 1 Siena Brain Investigation and Neuromodulation Lab (Si-BIN Lab), Department of Medicine, Surgery and Neuroscience, Neurology and Clinical Neurophysiology Section, University of Siena, 53100 Siena, Italy; lucia.mencarelli92@gmail.com (L.M.); romanellasara@gmail.com (S.R.); simone.rossi@unisi.it (S.R.); 2 Berenson-Allen Center for Non-Invasive Brain Stimulation, Beth Israel Deaconess Medical Center, Harvard Medical School, Boston, MA 02120, USA; 3 Neuroelectrics, Cambridge, MA 02139, USA; 08035 Barcelona, Spain; mariachiara.biagi@neuroelectrics.com (M.C.B.); ricardo.salvador@neuroelectrics.com (R.S.); giulio.ruffini@neuroelectrics.com (G.R.); 4 Human Physiology Section, Department of Medicine, Surgery and Neuroscience, University of Siena, 53100 Siena, Italy

**Keywords:** disorder of consciousness, brain networks, neuroimaging, neuromodulation, brain stimulation, network mapping, default mode network, connectivity, fMRI, tDCS, tES

## Abstract

Disorder of consciousness (DoC) refers to a group of clinical conditions that may emerge after brain injury, characterized by a varying decrease in the level of consciousness that can last from days to years. An understanding of its neural correlates is crucial for the conceptualization and application of effective therapeutic interventions. Here we propose a quantitative meta-analysis of the neural substrate of DoC emerging from functional magnetic resonance (fMRI) and positron emission tomography (PET) studies. We also map the relevant networks of resulting areas to highlight similarities with Resting State Networks (RSNs) and hypothesize potential therapeutic solutions leveraging network-targeted noninvasive brain stimulation. Available literature was reviewed and analyzed through the activation likelihood estimate (ALE) statistical framework to describe resting-state or task-dependent brain activation patterns in DoC patients. Results show that task-related activity is limited to temporal regions resembling the auditory cortex, whereas resting-state fMRI data reveal a diffuse decreased activation affecting two subgroups of cortical (angular gyrus, middle frontal gyrus) and subcortical (thalamus, cingulate cortex, caudate nucleus) regions. Clustering of their cortical functional connectivity projections identify two main altered functional networks, related to decreased activity of (i) the default mode and frontoparietal networks, as well as (ii) the anterior salience and visual/auditory networks. Based on the strength and topography of their connectivity profile, biophysical modeling of potential brain stimulation solutions suggests the first network as the most feasible target for tES, tDCS neuromodulation in DoC patients.

## 1. Introduction

Brain injury is one of the major causes of death and disability in the world [1]. As a consequence, several patients suffer from disorder of consciousness (DoC) (for specific statistics see [2]), a condition that can be divided into four states: (i) coma (patients are not able to spontaneously open their eyes and to be awakened even with strong sensory stimulation; [3]), (ii) vegetative state/unresponsiveness wakefulness syndrome (VS/UWS; patients are able to stay awake spontaneously or after stimulation, but they have no awareness of themselves or of the environment; [4,5]), (iii) minimally conscious state (MCS; patients show some behavioral evidence of consciousness; [6]) and (iv) patients emerging from MCS (EMCS; patients recover the ability to use objects in a functional manner, [7]). Patients may fluctuate between these different states until they fully recover consciousness, or may remain in a DoC state for years or permanently. Due to the strong impact of disease on patients and their caregivers, research aimed at improving diagnosis and therapy is a priority.

In recent years, consciousness has been defined as separated into two linearly correlated components: arousal and awareness [8]. Awareness can be divided in two distinct and negatively correlated networks: the ‘external awareness’ network, which includes bilateral fronto-temporo-parietal cortices, and the ‘internal awareness’ network, consisting of midline posterior cingulate cortex/precuneus and anterior cingulate/medial prefrontal cortices [9]. In support of this hypothesis, several neuroimaging and electrophysiological studies showed the presence of a structural and functional disconnection between these brain areas in DoC patients [10,11,12]. Specifically, deficits of cortico-subcortical (i.e., including the thalamus) and cortico-cortical connectivity have been proposed as one of the biological causes of DoC [13,14]. Resting-state fMRI (rs-fMRI) and PET studies suggest impaired inter-hemispheric connectivity in the ‘external awareness’ network [9], as well as in corticothalamic circuitry and the default mode network (DMN) in patients compared to healthy controls [15,16,17]. Moreover, a decrease in functional MRI resting-state low frequency fluctuations and regional voxel homogeneity [18] has been shown in DMN regions in patients with DoC. Historically, the DMN has been associated with conscious and self-related cognitive processes [19,20] such as inner or task-unrelated thoughts [21] and self-reflection [22], with a progressive decrease in the functional connectivity (FC) of DMN regions alongside the spectrum of consciousness [15,17,23,24,25].

Until now, both pharmacological and non-pharmacological therapies for DoC have been proposed, with contrasting results. Non-pharmacological interventions (reviewed in [26]) are divided into invasive (deep brain stimulation or vagal nerve stimulation) and non-invasive approaches (e.g., transcranial direct current stimulation—tDCS, repetitive transcranial magnetic stimulation—rTMS, transcutaneous auricular vagal nerve stimulation, low intensity focused ultrasound pulse and sensory stimulation program). Recent literature reviews [27,28] support the hypothesis that non-invasive brain stimulation (NIBS) is more successful than other therapies, but, considering the few studies available to date, these techniques are not yet officially recommended by clinical consensus groups. In particular, the dorsolateral prefrontal cortex (dlPFC) was identified as a better target for tDCS compared to the precuneus and motor cortex, due to its involvement in the cortico-subcortical network and to its strong connection with the thalamus and striatum, which are impaired in DoC according to the mesocircuit fronto-parietal model [29]. However, only two studies on tDCS provided class II evidence [28] and only in MCS patients, whereas for VS patients none of these approaches have provided group-level effects yet [30,31].

Although new therapeutic approaches seem to be beneficial for patients with DoC, the optimization of procedures and parameters should be the goal of future studies. So far, the stimulation target in patients with DoC has been chosen based on anatomical and/or physiopathological models. However, several neuroimaging studies revealed the disconnection between different brain networks in DoC (‘external awareness’ and ‘internal awareness’ networks), and also found that brain regions do not operate in isolation but rather continuously interact with each other [32,33,34]. Therefore, network targeting is becoming the main goal of neuromodulatory interventions and should be applied for patients with DoC as well. Several studies already showed the possibility of targeting an entire network by means of non-invasive brain stimulation (NIBS; [35,36,37]). In particular, Ruffini et al. (2014) developed an algorithm for the optimization of multielectrode tES that uses subject’s data (fMRI, PET, EEG or other data) to optimize personalized stimulation protocols in terms of electrodes position and stimulation intensity. This approach is applicable to all tES modalities (e.g. tACS, tDCS, tRNS) and had already been implemented successfully in healthy subjects [38,39,40] and patients [41,42,43].

In this study we present a quantitative meta-analysis with the aim of localizing the brain regions usually displaying altered activity both during external stimulation and at rest in patients with DoC, summarizing the fMRI and PET literature available to date. Network mapping was performed on brain regions resulting from the meta-analysis, pinning down the most relevant networks altered in DoC patients and also providing relevant details to inform future personalized therapeutic tES solutions.

## 2. Material and Methods

### 2.1. Literature Search

**Figure 1:**
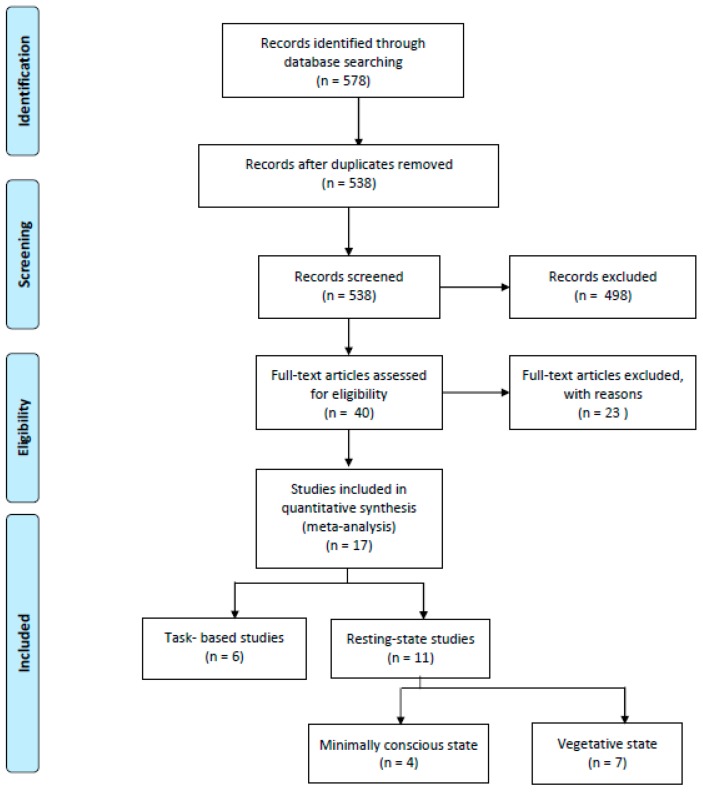
Literature search for the identification of relevant publications included in the ALE (activation likelihood estimate) meta-analysis (from [59]).

The literature search was carried out using PubMed and Google Scholar databases without temporal limitations. The following terms “disorder of consciousness”, “DoC”, “vegetative state”, “minimally conscious state”, “unresponsive wakefulness syndrome” were individually combined with “functional magnetic resonance imaging”, “positron emission tomography” and their acronyms. Following careful abstract screening, a total of 40 studies were chosen and scrutinized (Figure 1). We intentionally excluded (i) review papers, (ii) studies not mentioning any of the keywords in the abstract, (iii) studies not reporting fMRI/PET activations coordinates in MNI or Talairach space, (iv) studies not reporting activation foci in table format or reporting statistical values without corresponding coordinates, (v) studies that used predefined regions of interest (ROIs), (vi) studies reporting results obtained with small volume correction (SVC), (vii) studies not in the English language. The final selection included 17 studies reporting either fMRI or PET findings. For each study, the following information were retrieved: (i) number of participants, (ii) etiology; (iii) sex, (iv) mean age, (v) contrast, (vi) reference, (vii) foci, (viii) imaging modality (Table 1). Moreover, for task-related experiments, we also included the following information: task category, modality, and task type (Table 1). In particular, the etiology of patients considered in these studies is heterogeneous: traumatic brain injury (TBI), anoxic brain injury, cerebrovascular accident, hypoxic ischemic brain injury, hypoglycemia, subarachnoid hemorrhage, encephalitis, cardiopulmonary arrest, occlusion basilar artery, intoxication, stroke, cardiac arrest and aneurysm. Due to the limited literature, we decided to consider any cause of DoC, not just TBI. Moreover, the number of subjects for each study ranged from case reports [44,45], to large simple sizes (*n* = 27) [23,46]. The majority of the patients were male (225 on 341 total subjects), and the mean age was 44. Specific activation foci were collected and included in a quantitative activation likelihood estimation (ALE) analysis for the identification of brain regions most commonly reported as involved in DoC.

Two separate maps were created: (i) a task-based map including all the coordinates referring to fMRI/PET activations during specific tasks (6 studies; [44,45,46,47,48,49]), and (ii) a resting-state map considering all the coordinates referring to neural deactivation in DoC patients compared to healthy controls (11 studies; [23,25,50,51,52,53,54,55,56,57,58]). Furthermore, when selecting data for the resting-state ALE map, we divided fMRI/PET activations data for VS (including unresponsive wakefulness syndrome, UWS) and MCS patients, resulting into two distinct maps. Activation foci were extracted separately for MCS and VS/UWS patients for each study. Studies in which DoC patients were combined (e.g. VS and MCS) without reporting separate information for each condition were excluded from the analysis. Patients defined as in ‘coma’, were considered as in “VS” for the analysis. The final selection included 4 studies for MCS [50,53,55,57] and 7 studies for VS [25,52,54,55,56,57,58]. Given the limited number of studies focusing on each specific condition, more in-depth analysis was not feasible, therefore results must be considered exploratory in nature. Data on locked-in syndrome (LIS) and emerging minimally conscious state (EMCS) were not included in the analysis due to very limited literature.

### 2.2. ALE Maps Computation

The quantitative evaluation of spatial PET and fMRI patterns was carried out using the activation likelihood estimate (ALE) technique implemented in the GingerALE software v. 3.0 (www.brainmap.org) [60,61]. This software performs a statistical map indicating the set of significant voxels while considering the magnitude of the effect, the number of studies and the number of participants in each study. First, lists of coordinates were carefully checked for duplication of data across publications, in order to avoid artefactual inflation of a given foci significance. The coordinates reported in the Talairach space were transformed into MNI coordinates through the tal2mni algorithm implemented in GingerALE. The reported foci of activation for each study were modeled as Gaussian distributions and merged into a single 3D volume. Equally-weighted coordinates were used to form estimates of the probability of activation for each voxel in the brain, using an estimation of the inter-subject and inter-study variability, rather than applying a priori full-width half maximum (FWHM) kernel. Therefore, the number of participants in each study influenced the spatial extent of the Gaussian function used. We first modeled the probability of activation over all studies at each spatial point in the brain, returning localized “activation likelihood estimates” or ALE values. Values were then compared to a null distribution created from simulated datasets with randomly placed foci to identify significantly activated regions (permutations test = 1000 run). A family-wise error (FWE) correction both at cluster level and voxel level (*p* < 0.001 for cluster-formation; *p* < 0.05 for cluster-level inference) were applied. ALE maps were visualized using MRICronGL on an MNI standard brain.

### 2.3. Neuroimaging Analysis

#### 2.3.1. MRI Dataset

In order to perform network mapping, a fMRI dataset collected at Beth Israel Deaconess Medical Center was used. The dataset included 187 healthy participants (mean age 29 years, range 21 to 49, SD = 12; mean education 15 years, range 11 to 23, SD = 3). Neuroimaging data were acquired on a 3.0 T General Electric (GE Medical Solutions, Erlangen, Germany). For each subject, a three-dimensional T1-weighted MPRAGE image was acquired in the axial plane (TR/TE 2500/3.5 ms; 192 slices; slice thickness 1 mm; flip angle 8°; voxel size 1.0 × 1.0 × 1.0 mm). Resting-state fMRI data were collected using T2-weighted BOLD images (TR/TE 2500/30 ms; 38 interleaved slices; slice thickness 3 mm; 260 volumes; flip angle 80°; voxel size 3.0 × 3.0 × 3.0 mm). Participants were asked to keep their eyes open in the scanner while fixating on a cross-hair without focusing on any topic.

#### 2.3.2. fMRI Preprocessing

Preprocessing of the functional images was carried out using SPM8 (Wellcome Department of Cognitive Neurology, Institute of Neurology, University College London; http://www.fil.ion.ucl.ac.uk/spm/) within the MATLAB scientific computing environment (http://www.mathworks.com, MathWorks, MA, USA). The first five volumes of functional images were discarded for each subject to allow for steady-state magnetization. EPI images were then stripped of skull and other non-cerebral tissues, slice-timed using interleaved descending acquisition, manually realigned, and subsequently resliced. Structural images were co-registered to the mean volume of functional images and subsequently segmented using the NewSegment routine in SPM8. A hidden Markov random field model was applied in order to remove isolated voxels. Moreover, to obtain a more accurate spatial normalization we applied the SPM8 DARTEL (diffeomorphic anatomical registration through exponential lie algebra) module, creating a customized gray matter template from all subjects’ segmented images [62]. A nonlinear normalization procedure to the Montreal Neurological Institute (MNI) template brain, and voxel resampling to an isotropic 3 × 3 × 3 mm voxel size, were then applied. We removed linear trends to reduce the possible influence of the rising temperature of the MRI scanner. All functional volumes were band-pass filtered between 0.01 and 0.08 Hz to reduce low-frequency drifts. Finally, we controlled the potential contribution of nuisance sources of variability to grey matter BOLD time courses by regressing out the head motion parameters as well as the signal derived from four regions of interest (ROIs) placed in the white matter and cerebro-spinal fluid. This approach has been shown to significantly enhance within-subject and test-retest reliability [63].

#### 2.3.3. Seed-Based Functional Connectivity

To characterize the functional connectivity pattern of each region resulting from the ALE meta-analysis, a seed-based connectivity analysis was conducted by extracting the average BOLD time course from all the voxels included in a given resting-state map (e.g., altered rs-fMRI connectivity in MCS patients). Subsequently, we correlated the signal from each map with the remaining voxels in the rest of the brain, resulting in a 3D weighted volume where each voxel value represents the correlation coefficient between its BOLD activity and that of the seed map of interest. Results were computed applying a voxel-level threshold (*p* < 0.001, false discovery rate-FDR-corrected) and cluster size correction (*p* < 0.001, family-wise error-FWE-corrected).

### 2.4. Clustering Analysis

Given the different significant regions identified by the ALE analysis, the presence of similar connectivity alterations was also tested by comparing their respective seed-based connectivity maps via a functional clustering algorithm (Matlab 2016b, The Mathworks). The algorithm identified similarity in the cortico-subcortical functional maps derived from each ALE region, assigning them to *N* clusters based on their profile (accounting for both topography and sign of connectivity values). The analysis allowed us to reduce the number of potential networks altered in DoC, allowing us to focus on identifying and testing possible tES solutions to enhance connectivity in patients.

### 2.5. Similarity Index

Once the main functional connectivity clusters were identified, the functional maps belonging to the same cluster were averaged together, resulting in a whole-brain connectivity map depicting a major network altered in DoC. In order to characterize the functional profile of each resulting network, functional labeling was performed by looking at the spatial similarity of each network map and those of known RSNs using a weighted variant of the DICE coefficient (weighted dice coefficient, wDC) [64]. RSNs were defined following the parcellation scheme by Shirer et al., (2012), reporting 12 non-overlapping maps of different networks: default mode (DMN), right and left executive control (RECN, LECN), dorsal attention (DAN), anterior and posterior salience (AS, PS), basal ganglia (BG), language (LANG), high and primary visual (HVIS, PVIS), auditory (AUD), and somatosensory (SM) [65]. Over the last 15 years, different research groups have applied various approaches for extracting and labeling RSNs. In this study, we considered the AS as the network including the bilateral insula (mostly referring to its anterior part) and the dorsal anterior cingulate cortex (dACC). However, according to the work by Dosenbach et al. (2007), the same network, with the inclusion of two anterior frontal regions corresponding to Brodmann area 9/10, is known as the cingulo-opercular network. The same applies to the LECN and RECN, indicating two lateralized networks resembling the fronto-parietal control network as originally described by the same group [66,67]. Both definitions, with the additional distinction of a left and right component in Shirer et al. (2012), refer to a network involved in cognitive control, with a specific involvement in control initiation, flexibility, and modulation of response to feedback. In addition, the AN identified here reflects the ventral and dorsal attention network proposed by Corbetta et al. (2008), with no differentiation between a dorsal (including bilateral parietal lobe, frontal eye fields and, to a lesser degree, parieto-occipital regions) and a ventral part (i.e., more frontal, including regions of the inferior and middle frontal gyrus) [68]. Moreover, we decided to group together vDMN, dDMN, and precuneus in a single network (DMN). Finally, another classification has been proposed by Yeo et al. (2011), including multiple labeling solutions acknowledging the existence of 7 or up to 17 resting-state fMRI networks. The main difference with respect to the work by Shirer et al. (2012) concerns the labeling of a subset of prefrontal regions, classified as part of the FPCN by Yeo et al. (2011) instead of AN [65,69].

Importantly, the comparison of weighted, unthresholded connectivity maps for each DoC network and RSN map at the single voxel level requires considering not only spatial similarity, but also similarity of the connectivity sign (i.e., positive and negative connectivity). Therefore, the similarity index was obtained by computing the product of each voxel’s value across two maps (e.g., voxel j in the DoC and DMN maps), resulting in a map where positive values represent voxels with the same sign in both maps (i.e., positive connectivity in both DoC and DMN), while negative ones represent opposite signs (i.e., positive connectivity value in voxel j in DoC, negative in DMN). As a result, the magnitude of the similarity index represents the similarity of connectivity strength in any two given maps (expressed as wDC). This procedure allowed to identify similar connectivity profiles between the main networks altered in DoC (as resulting from functional clustering of regions showing altered activity during fMRI or PET imaging) and known RSNs, thus providing insight about the function and meaning of large scale networks altered in DoC.

## 3. Results

### 3.1. ALE Meta-Analysis

The results of the ALE meta-analysis are available for download as a nifti. nii volumetric file at (www.tmslab.org/santalab.php). The maps include network-level volumes representing the entire set of regions presented in the following paragraphs. Detailed information on the anatomical localization of each significant regions and the relative statistics is reported in dedicated figures and tables. A more in-depth discussion about the meaning of the patterns identified, as well as the role of specific regions, is provided in the discussion section.

### 3.2. Task-Based Map

**Figure 2:**
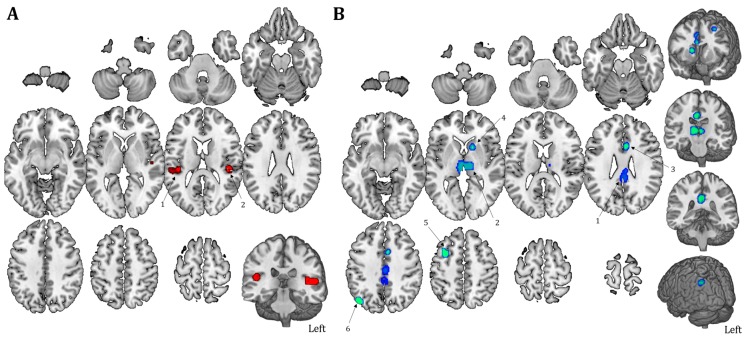
Task- and resting state-related activation map. (**A**) Temporal lobes are strongly activated when DoC patients performed a task (in red). (**B**) Brain hypoactivation in both internal and external awareness networks in patients compared to healthy controls during rs-fMRI (resting-state functional magnetic resonance imaging) is shown (blue). The ALE region numbers are reported.

The resulting map and coordinates of the neural activity patterns during active or passive tasks execution are reported in Table 2 and Figure 2A. The map includes 2 regions showing a very specific activation of the bilateral temporal lobes (BA 41, MNI coordinates of main clusters: x = −52, y = −30, z = 10; x = 46, y = −28, z = 12), without contribution of any other region.

### 3.3. Resting-State Maps

**Figure 3:**
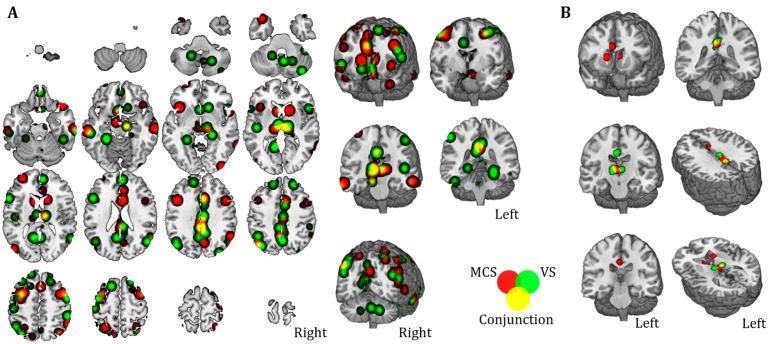
Similarities and differences between MCS (Minimally Conscious State) and VS. Brain hypoactivation during resting-state is shown for MCS (red) and VS patients (green), as well as for overlapping regions (yellow). The map shows the qualitative overlap without any statistical threshold (panel A), as well as with a family-wise error (FWE) correction both at the cluster level and voxel level (*p* < 0.001 for cluster-formation; *p* < 0.05 for cluster-level inference, panel B).

Figure 2B and Table 3 show the pattern of deactivation in patients with DoC during resting-state compared to healthy subjects. Results on the entire sample of DoC patients include 6 separate regions highlighting the involvement of cortical areas (frontal and parietal areas in particular: BA 6 and BA 39, MNI coordinates of main regions: x = −36, y = 6, z = 54; x = −44, y = −70, z = 40) and subcortical regions (e.g., cingulate gyrus; BA 31; MNI coordinates of main regions: x = 0, y = −36, z = 32; caudate; MNI coordinates of main regions: x = 14, y = 14, z = 8; and thalamus; MNI coordinates of main regions: x = 8, y = −16, z = 6). Similarities and differences in resting-state connectivity of MCS and VS patients are also shown (Figure 3, Table 4), specifically referring to regions of decreased fMRI activity in DoC patients. Major connectivity alterations are visible in the thalamus for both VS and MCS. Interestingly, MCS patients present alteration of more anterior subcortical structures (i.e., right and left caudate nuclei), whereas VS patients display a significant decrease in connectivity in more posterior structures (i.e., posterior cingulate cortex).

### 3.4. Functional Connectivity Mapping

**Figure 4:**
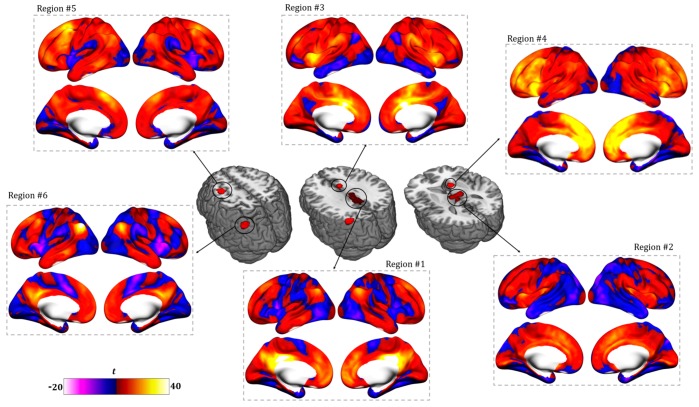
Network mapping. Positive and negative functional connectivity profile for each ALE region is shown. Surface representation underlies the high similarity between the functional connectivity (FC) of regions #1, #5 and #6 and between the FC of regions #2, #3 and #4.

In order to better illustrate the spontaneous functional connectivity of the regions resulting from the resting-state map, a seed-based analysis was run on a database of healthy subjects previously collected at Beth Israel Deaconess Medical Center (BIDMC) in Boston (MA, USA). Figure 4 shows the functional connectivity profile of each region separately. In particular, the functional connectivity profile between the only two cortical regions identified at the ALE analysis (region #5 and #6) and the posterior cingulate gyrus (region #1) resemble the DMN and FPN, while the functional connectivity of subcortical areas, specifically the thalamus (region #2), anterior cingulate gyrus (region #3) and caudate nuclei (region #4), resembles the anterior and posterior salience networks (AS, PS).

**Figure 5:**
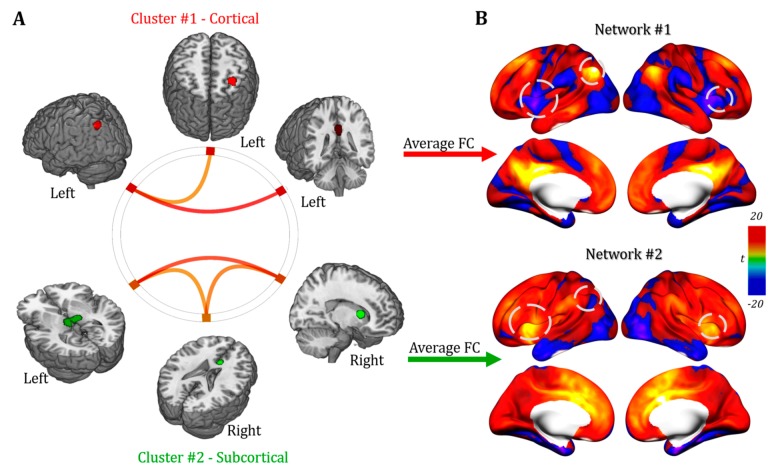
**Functional** cluster mapping. (**A**) A strong similarity within the functional connectivity profile of cortical regions (Cluster #1, red) and subcortical regions (Cluster #2, green) was highlighted by the functional clustering analysis. (**B**) Resulting average functional connectivity maps characterizing cortical and subcortical clusters (i.e., Networks #1 and #2) show similar topography in the anterior prefrontal cortex but partially negatively correlated profiles in parietal and ventrolateral prefrontal cortices (dashed circles).

Furthermore, as shown in Figure 5A, clustering analysis (*p* < 0.05, FDR at single voxel level; *p* < 0.05 NBS correction at whole network level) reveals a quantitative estimate of the positive connectivity between cortical nodes (middle frontal gyrus and angular gyrus) and the posterior cingulate gyrus, as well as a positive correlation among the three subcortical nodes (thalamus, caudate nuclei and anterior cingulate gyrus). As expected, the functional clustering algorithm grouped the six maps into two main clusters (Cluster #1 and #2) resembling cortical nodes in the first one and subcortical structures in the second one (Figure 5A). The resulting two connectivity maps obtained by averaging the connectivity maps of cortical and subcortical regions separately displayed different topography (Network #1 and #2 hereafter; Figure 5B).

### 3.5. DoC Networks and RSNs

**Figure 6:**
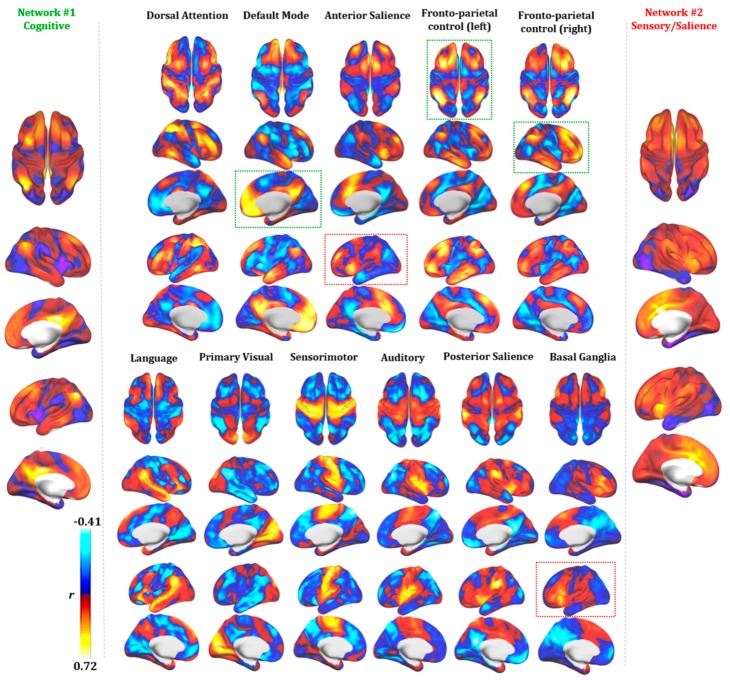
Functional connectivity profile of the two identified DoC networks. A visual comparison of seed-based connectivity maps for DoC networks (Network #1 and #2) and major Resting State Networks (RSNs) is shown. Red and blue colors represent the intensity and polarity of connectivity between each network and the rest of the brain. At the qualitative level, Network #1 (cognitive) resembles the fronto-parietal control network (right and left FPCN) and the Default Mode Network (DMN) is highlighted (green dotted lines). Qualitative similarity is also present for Network #2 (sensory/salience) and the anterior salience and basal ganglia networks (red dotted lines). Connectivity is expressed as the correlation coefficient between the average Blood Oxygen Level-Dependent (BOLD) signal extracted from each map and that of any other voxel in the brain.

Cortical and subcortical maps for multiple RSNs were computed in order to provide a qualitative comparison with DoC Network #1 and #2. As shown in Figure 6, a qualitative spatial similarity analysis suggests Network #1 mostly resembles the right and left fronto-parietal control networks (FPCN), given its strong activation in prefrontal and parietal areas, as well as the DMN due to high connectivity in the precuneus. The FPCN is usually associated with cognitive control, with a specific involvement in control initiation, flexibility and modulation of response, whereas DMN is mostly involved in self-related and internal control. The similarity between Network #1 and these two cognitive networks led us to label Network #1 as a ‘Cognitive’ network altered in DoC. Network #2, instead, showed high similarity with the anterior salience (AS) and basal ganglia (BG) networks, suggesting more specific matching in ventrolateral prefrontal and parietal cortices. These two networks are involved in top–down control over sensory and limbic regions, as well as in the integration of external sensory information with internal emotional and bodily states [70,71]. Because of the similarity between Network #2 and RSNs involved in salience and sensory perception, we labeled Network #2 as ‘Sensory/Salience’.

**Figure 7:**
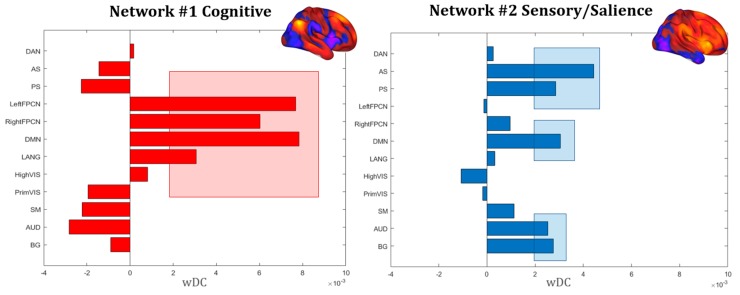
Similarity coefficient. Weighted DICE coefficients (wDC) for every RSN are shown, confirming the higher similarity between Network #1 and DMN, left and right FPCN and LANG, as well as the dissimilarity in connectivity profile with the AS, PS, auditory, visual and somatomotor networks. The opposite pattern was identified for Network #2, showing higher similarity for networks involved in sensory perception (AUD, SM, BG) and salience (AS, PS). Red and blue rectangles underline the higher significant overlap between the RSNs and DoC networks. Note: default mode network (DMN), right and left fronto-parietal control networks (right and left FPCN); dorsal attention network (DAN), anterior and posterior salience networks (AS, PS), basal ganglia network (BG); language network (LANG); high and primary visual networks (HighVIS, PrimVIS); precuneus network (Precuneus); auditory network (AUD); somatosensory network (SM).

Quantitative similarity analysis confirmed the pattern (Figure 7), while also underlining a similarity between Network #1 and the language network (LANG), as well as between Network #2, DMN and the auditory network (AUD). Interestingly, the two networks display a complementary pattern in terms of their loading on known RSNs, further confirming the different nature of the two separate clusters identified via functional clustering of ALE regions.

**Figure 8:**
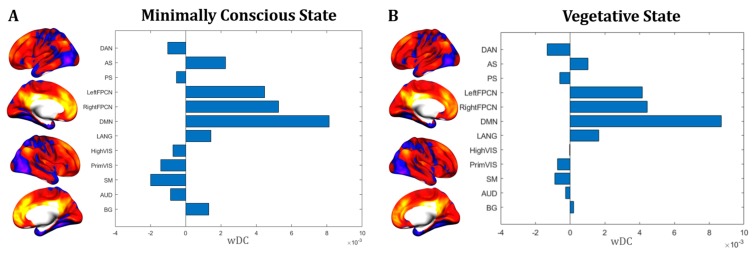
MCS, VS and RSNs. The functional connectivity profile and the similarity coefficient with RSNs is shown separately for MCS (**A**) and VS (**B**). A similar network profile was identified for both MCS and VS patients, with major overlap with DMN and FPCN as shown for Network #1.

Additionally, the same analysis performed by splitting MCS and VS connectivity maps shows a similar pattern for both groups, which is mostly comparable to Network #1—Cognitive (Figure 8).

### 3.6. Biophysical Modeling Results

The optimal multichannel montages to target the aforementioned networks on an MRI-derived realistic head model can be determined by optimization algorithms. These algorithms require the creation of a signed target map out of the network, indicating the importance of each network area in the optimization, as well as the desired stimulation effect (excitation/inhibition). Detailed information about these methods is reported in the supplementary material.

**Figure 9:**
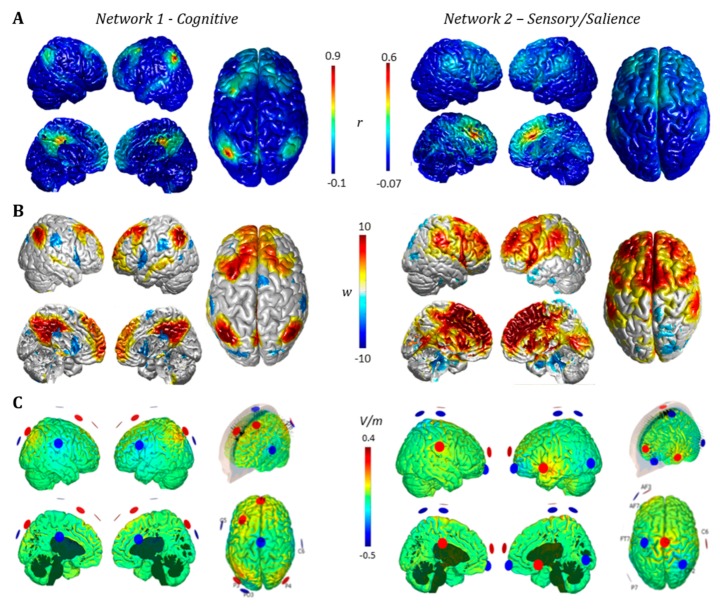
Multichannel transcranial electrical stimulation (tES) optimized montages. (**A**) Functional connectivity profile for Network #1 and #2, mapped onto the cortical surface. (**B**) Weighted target maps for both networks. The values in the scale correspond to the weights multiplied by the sign of E_n_^target^ to display excitatory and inhibitory areas. For Network #1, correlations higher than 0.4 were assigned to excitation with maximum weight *w_max_* = 10, whereas lower than −0.06 were assigned inhibition with lower negative weight *w_min_* = 5. For Network #2, the threshold value for excitation was set to 0.15 and for inhibition to −0.04. (**C**) Optimized montages for Networks #1 and #2. Both solutions involve 8 electrodes, delivering a total maximum current of 4 mA. Anodes are shown in red, cathodes in blue; arrows represent current density.

Figure 9 shows the result of multichannel tES montage optimization to promote the activation of positively correlated FC areas in DoC patients. The signed weight maps used for the two optimizations are shown in Figure 9 (panel b) for Network #1-Cognitive (left) and Network #2-Sensory/Salience (right). Because the maximum negative correlation values *r* for both networks are, in absolute, lower than the maximum positive ones (for Network #1 r_min_ = −0.118; r_max_ = 0.982; for Network #2 r_min_ = −0.102; r_max_ = 0.839), we assigned a maximum weight of w_min_ = 5 to the areas to inhibit, and a maximum weight of w_max_ = 10 to the areas to excite.

For Network #1, values of r > 0.4 and r < −0.06 were clipped to w_max_ and w_min_ respectively; values |r| < 0.045 were considered not significant to target and set to no-stimulation with low weight w_0_ = 2. For Network #2, we mapped r > 0.15 to w_max_, r < −0.04 to w_min_, and |r| < 0.02 to w_0_. For both networks, intermediate negative correlation values were linearly rescaled between w_0_ and w_min_, and positive ones between w_0_ and w_max_. We observed that the maps thus created represent a reasonable translation of the cortical FC correlation information in terms of the target network, as they retain and emphasize only the areas with the strongest positive and negative correlation, with consistent significance. In particular, we observe that for Network #2, the FC correlation between cortical areas and seeds is much lower in the external surface than in the midsagittal region (Figure 9, panel A, right). However, since tES can induce higher E_n_ on the external cortex than on the internal surfaces, the weight map enhances the importance of the former as a target for the stimulation as the preferential venue to reach the deep cortical seed.

The optimized montages are shown in Figure 9 (panel c) for Network #1 and #2. Both montages involve 8 electrodes, delivering a total injected current of approximately 4 mA (max current per electrode was limited to 2.0 mA). For Network #1, the montage comprehends C6: −831 uA, CZ: -741uA, FC1: 759uA, FC5: -1054uA, FZ: 826uA, P3: 1576uA, P4: 837uA and PO3: -1372uA. For Network #2, it comprehends: AF3: 829uA, AF7: -1402uA, C1: -710uA, C6: 398uA, CP2: -1257uA, CZ: 1650uA, FT7: 1121uA and P7: -629uA. Given the current constraints, these solutions represent the best fit of the E_n_ to the target maps obtained from FC correlation values. Moreover, using only 8 electrodes, they reach respectively 95% and 89% of the optimal fit value using a full electrode cap with 64 channels. For Network #1, the optimized montage induces an average normal electric field on the correlated areas, set to excitation, of <E_n, ex_ > = 0.014V/m and on the anticorrelated areas, set to inhibition, of <E_n, in_ > = −0.016 V/m. The montage for Network #2 induces <E_n, ex_ >= 0.007 V/m on the areas set to excitation, and <E_n, in_ >= −0.003 V/m on the areas set to inhibition. Results suggest that biophysical optimization of Network #1 achieves stronger e-fields compared to Network #2 on average, therefore suggesting the former as the most suitable target for network stimulation in DoC.

## 4. Discussion

In the present work, we reviewed all the studies reporting fMRI or PET activity in DoC patients, in order to provide a set of activation/deactivation maps related to task-evoked or resting-state activity. We expanded these findings by mapping the functional connectivity profile of each identified region, and by calculating the similarity coefficient between functional networks altered in DoC patients and canonical RSNs maps. In the following paragraphs we discuss our results and their relevance for possible new therapeutic applications in DoC, including personalized transcranial electrical stimulation (tES) solutions aimed at rebalancing altered network dynamics.

### 4.1. Brain Activations and Deactivations in DoC

The brain activation pattern reported during tasks in DoC is mostly restricted to the bilateral temporal cortices, reflecting the use of passive auditory tasks during fMRI in most of the considered studies. The task materials and presentation modalities were varied in the articles included in the meta-analysis. An example of active task during fMRI was: “imagine navigating your home” [47]; whereas, during the passive task participants heard long spoken narrative regarding everyday events [44]. Despite this dissimilarity, it was not possible to compute two different maps (active/passive task), as only one study used an active task paradigm. Differently from a similar analysis done by [72], we did not find activity in the bilateral orbito-frontal and frontal gyrus. However, our results are comparable with theirs when comparing results obtained with the same cluster correction approach, i.e., family wise error (FWE, see Table S2 in [72]). As suggested by [73], cluster-level FWE correction is the most reliable approach to control for false positives [73]. Moreover, following the same recommendation, we also used a more conservative mask size compared to [72].

As for resting-state results, diminished activity in the posterior and anterior cingulate cortex, thalamus, angular gyrus, and prefrontal cortex were found, in line with previous literature [74]. Subcortical regions mostly matched with the internal awareness network, also defined as the network involved in mental processes (i.e., mind wandering, daydreaming, mental imagery) without the requirement of external stimuli [9]. On the other hand, cortical regions (middle frontal gyrus and angular gyrus) resemble the external awareness network, typically involved in conscious perception of environmental stimuli and in goal-directed behavior [9]. Results also confirm the central role of the thalamus in consciousness [74], as well as the necessary link between the cortex and thalamus for conscious perception [49,75], with thalamic lesions classically leading to global loss of consciousness [76]. Moreover, considering the analysis conducted separately for MCS and VS patients, the central role of the thalamus stands out again. As shown in Figure 4 and in line with a previous study [74], in VS patients both thalami seem less activated at rest, whereas in MCS patients only the right thalamus is impaired compared to healthy controls. Additionally, from a qualitative point of view, the main difference between MCS and VS resides in a differential alteration of the internal network, with a more anterior impairment in MCS compared to a more posterior deactivation in VS patients. These results are also in line with a previous study by [17] showing that only medial parietal regions are sensitive to differences in functional connectivity between MCS and VS [17].

Overall, during a passive task, a specific pattern of activity in the temporal lobe seems present, whereas reduced activity during rs-fMRI is observed mostly in subcortical structures of the internal network. However, a widespread reduction of activity in DoC patients compared to healthy controls is also visible in cortical areas (i.e., middle frontal gyrus and angular gyrus) of the external network [9].

### 4.2. Network Mapping

The functional connectivity analysis conducted on ALE regions based on resting-state data shows the involvement of multiple functional networks possibly responsible for different clinical characteristics of DoC. Our results suggest that areas of hypoactivation in DoC patients belong to two main networks: Network #1, resembling networks related to high-order cognitive processing and executive functions (right and left FPCN) and Network #2, resembling networks involved in salience and sensory perception (AS and BG). Moreover, both networks display clear overlap with the DMN, as already pointed out by several studies [17,51,74].

In particular, quantitative similarity analysis between Network #1 and RSNs suggests higher similarity with right and left FPCN. These networks are mostly related to cognitive processes, such as control of attention allocation, abstract reasoning and flexibility. Moreover, they are highly involved in fluid intelligence [77] and executive functions (updating, switching and inhibition, [78]) and play a relevant role in mediating the dynamic balance between DMN and DAN [79]. The impairment of areas strongly related to the FPCN in DoC patients was previously pointed out by [80]. Using graph-theory analysis of BOLD data, the authors reported that fronto-parietal network proprieties are altered in several regions associated with conscious processing. Additionally, the middle prefrontal cortex (a central hub in the FPCN) has been associated with emotional balance, response flexibility and self-knowing awareness, processes impaired in DoC patients.

Despite Network #2 showing less similarity with other RSNs compared to Network #1, we found an interesting resemblance to the AS and PS networks. These networks are usually involved in monitoring and maintaining performance during a task [81,82], as well as playing a role in cognitive control and error detection due to their involvement in top-down control over sensory [83] and limbic regions [84]. Impairment in these networks could be driven by the loss of thalamic activity: anatomically the thalamus is well connected to the salience network since the interoceptive signals pass through the autonomic afferent nuclei and the thalamus before reaching the insula and are then dispatched to other cortical areas of the salience network where signals are integrated and used to coordinate other large scale cortical networks [70].

Moreover, the well-known similarity between both DoC networks and DMN is not surprising considering that the DMN has been linked to self-related and internal processes, such as stimulus- independent thoughts [21], mind-wandering [85], social cognition [86], introspection [87], monitoring of the ‘mental self’ [88] and integration of cognitive processes [89] (for a review see: [90]).

The hypoactivity in brain areas that are functionally related to these networks could be linked to patients’ deficit in perception of external stimuli, maybe not at the primary level (since the task-based ALE map shows an activation in the temporal cortex probably related to auditory tasks), but in the connections between primary areas and associative cortices, responsible for cognitive behavior, motor planning, memory and higher cortical functions.

Finally, network mapping reveals very similar functional connectivity patterns between MCS and VS, with a main involvement of DMN and bilateral FPCN, as previously observed for Network #1. Unfortunately, due to the small number of available studies, our results are not strong enough to detect any significant difference in VS and MCS functional connectivity profile. Future studies should focus on exploring the potential for imaging biomarkers to differentiate VS and MCS patients, also leveraging network-level alterations identified by means of combined TMS-EEG studies [26,91,92].

### 4.3. Potential Therapeutic Interventions

An effective treatment for DoC has not been identified yet, and the clinical management of these patients remains very challenging. Invasive and non-invasive therapeutic interventions have been proposed with inconsistent results [26]. With regard to noninvasive brain stimulation, several studies have shown the efficacy of tDCS over the DlPFC in improving patients’ responsiveness to external stimuli after both single [93] and repeated stimulation sessions [94,95]. Although available studies reported a beneficial tDCS effect in MCS compared to VS patients, even within MCS patients, high variability in response to the treatment was present and any effect was not strong enough to impact patients’ clinical status [31]. Such heterogeneity may be explained by single site stimulation and therefore an inability to produce a meaningful whole brain effect. Following this rationale, in a recent study [43], the fronto-parietal network was stimulated using tDCS. However, results showed that only 30% of MCS patients positively responded to stimulation. Crucially, in this study the stimulation targets were not chosen based on functional connectivity maps [43].

In an attempt to move the field towards image-guided, network-based stimulation in DoC, here we leveraged results of network mapping to test two tES montages designed to modulate cortical and subcortical regions identified via the ALE analysis. The approach considers functional connectivity maps as a target for stimulation montage optimization, selecting number and location of the electrodes as well as stimulation intensity according to weighted distribution of connectivity values. Such an approach allows one to indirectly target subcortical structure using transcranial cortical stimulation by leveraging cortical projections of deep structures, constituting a potential alternative for the treatment of pathologies currently addressed by DBS [96].

In the optimized montage solution proposed, and in agreement with the connectivity profiles of Network #1 and #2, lower priority has been assigned to the negative correlated areas than to the positive correlated ones. Therefore, a stronger excitatory than inhibitory effect is expected for both target networks. The results indeed reveal that in both montages the electrodes delivering the highest currents are anodes, placed over the areas to excite: the parietal lobes for Network #1 are targeted by P3/P4, whereas the superior frontal gyrus for Network #2 is addressed by Cz and AF3. However, the overall excitatory effect is larger than the inhibitory one only in Network #2, whereas in Network #1 the two effects are comparable. Moreover, both excitatory and inhibitory effects are stronger in Network #1 than in Network #2. These differences result from the combination of head anatomy and current constraints with network topology. In Network #2, a large portion of highly relevant areas to be excited lie on the midsagittal cortex, which is rather difficult to reach with scalp electrodes due to the limited penetration depth of the current. In Network #1, instead, important patches are also located on the lateral cortex, where, with the current less attenuated, the excitatory effect can be higher. On the other hand, negatively correlated areas lie only on the cortical surface, and they are larger and have slightly higher (negative) weight in Network #1 than in Network #2. Consequently, the inhibiting electric field on the negative correlated areas is larger for Network #1.

In short, the montage created for targeting Network #1 induces stronger effects, with a good balance between excitation and inhibition. This montage solution could help in reaching deep brain structures by stimulating multiple cortical areas functionally connected to DMN and FPCN, whereas the solution proposed for targeting Network #2 seems to be less efficient possibly due to the fact that subcortical regions altered in DoC do not display a strong functional correlation with cortical structures.

### 4.4. Limitations of the Study and Future Directions

The aim of this study was to show the brain activation/deactivation pattern typically involved in DoC patients during task-evoked activity or resting-state, and the similarity of these nodes to known RSNs. Even though we consider our ALE maps the most accurate representation of available literature, potential publication biases should be considered. Based on a recent study [73], the importance of finding a balance between homogeneity and power has to be considered. ALE maps based on a small number of studies are more affected by study heterogeneity [97], while at the same time focusing on a specific topic/task (and therefore a smaller number of studies) is also important to provide specific contribution to the field of interest. In this work we decided to focus on alterations of brain activity in DoC as a whole, complying with the criteria of homogeneity and including patients with DoC of any cause. Unfortunately, due to the limited literature available so far, we could not create specific ALE maps for every source of DoC, which should remain the ultimate goal of this type of work.

Moreover, a crucial missing element in our analysis is a characterization of the increase in neural activity observed in DoC compared to healthy controls. However, the number of studies reporting this information is very limited and it is not possible to compute reliable ALE maps. Future studies should investigate this aspect, in order to understand whether the increased brain activity in DoC could represent a compensatory mechanism and possibly an alternative therapeutic target.

Additionally, a few methodological issues in the study of rs-fMRI connectivity in DoC patients should be considered. DoC patients often present severe and heterogeneous brain damage: anatomical defects alter FC estimation as well as confound estimation of current distribution during brain stimulation [98]. Moreover, group-level functional connectivity analysis is usually performed by normalizing individual MRI data to a reference space (e.g., MNI), inevitably losing spatial resolution and individual features. Even though the approach presented in our study is a valuable step forward in the direction of identifying pathology-specific NIBS montages, future studies should involve modeling of individual MRI-fMRI data and personalized NIBS solutions for each patient.

## 5. Conclusions

Network mapping performed on brain regions resulting from our meta-analysis suggests a link between brain regions altered in DoC patients and two sets of brain networks representing internal mentation/cognitive control/mind wandering and sensory/salience processing, respectively. Based on biophysical modeling of network alterations, the most effective brain stimulation solution for patients with DoC involves stimulation of a network resembling the DMN and FPCN, promoting the value of network mapping and personalized montage optimization in future DoC studies.

## Figures and Tables

**Table 1:** List of studies considered in the meta-analysis. Sample size, etiology of DoC (Disorder of Consciousness), gender and age of sample, reference MRI space, number of foci, imaging modality and experimental conditions are shown. Additional information about the specific experimental paradigm are reported for task-based studies. Patients labeled as in a “coma” were relabeled as Vegetative State (VS); studies specifically comparing Locked-in Syndrome (LIS) and Emerged from Minimally Conscious State (EMCS) patients were not included in the analysis due to small sample size.

Paper	Subjects	Etiology	Sex (F)	Age (Mean)	Reference	Foci	Imaging Modality	Contrast	Task Category	Modality	Task Type
Task-based studies											
**Fernández-Espejo et al., 2010** [44]	1 UWS	TBI	0	48	MNI	2	fMRI	For > back; listen > silence	passive	auditory	sentences listening
**Liang et al., 2014** [47]	5 (2UWS, 3 MCS)	TBI	2	42.8	MNI	10;6;11;4	fMRI	Listen > rest; Navigation > rest; counting > rest; face > rest	active and passive	auditory	spoken sentenses and motor/mental imagery
**Monti et al., 2013** [45]	1 MCS	TBI	---	---	MNI	33	fMRI	no contrast	passive	auditory	visual stimulation
**Owen et al., 2002** [48]	3 UWS	acute febrile illness; TBI; cardiorespiratory arrest	3	28	MNI	8	PET	visual stimulation; familiar face perception; speech perception	passive	visual and auditory	visual stimulation
**Marino et al., 2017** [46]	50 (23 UWS, 27 MCS)	TBI, anoxic brain injury; cerebro-vascular accident		50	MNI	12	fMRI	no contrast	passive	auditory	sentences listening
**Laureys et al., 2000** [49]	5 UWS	hypoxic origin	3	44	TAL	4;8	PET	no contrast; DOC<HC	passive	auditory	click
Resting-State Studies											
**Bruno et al., 2010** [58]	10 UWS	cronic post-anoxic enephalopathy	2	44.3	MNI	16	PET	DOC < HC			
**Bruno et al., 2012** [50]	27 MCS	anoxia; TBI; subarachnoid hemorrhage; encephalitis; hypoglycemia; cerebro-vascular accident	10	45	MNI	40	PET	DOC < HC			
**Demertzi et al., 2014** [23]	53 (5coma, 24 UWS, 24 MCS)	brain insult	23	50	MNI	50	fMRI	DOC < HC			
**He et al., 2014** [51]	12 (9 UWS, 3MCS)	TBI; cerebro-vascular accident; anoxic brain injury	4	44.7	MNI	8;8	fMRI	DOC < HC; DOC > HC			
**Kim et al., 2010** [52]	12 UWS	anoxic brain injury	5	41.7	MNI	4;3	PET	DOC < HC; DOC > HC			
**Kim et al., 2013** [53]	17 MCS	hypoxic-ischemic brain injury	8	40.5	MNI	16;5	PET	DOC < HC; DOC > HC			
**Koenig et al., 2014** [54]	17 coma	cardio-pulmunary arrest	3	55	TAL	3	fMRI	DOC < HC			
**Nakayama et al., 2006** [55]	30 (17 UWS, 13 MCS)	TBI	11	30	TAL	13;10	PET	DOC < HC			
**Norton et al., 2012** [25]	13 (11 irreversible coma, 2 reversible coma)	cardiac arrest	5	66.3	MNI	16	fMRI	DOC < HC			
**Soddu et al., 2016** [56]	15 (11 UWS, 4 LIS)	anoxia; cerebro-vascular accident; TBI; hypoglycemia; occlusion basilar artery	10	45	TAL	17	PET/fMRI	DOC < HC			
**Thibault et al., 2012** [57]	70 (24 UWS, 28 MCS, 10 EMCS,8LIS)	TBI; cardiac arrest; stroke; intoxication; anoxia; hydrocephali; meningitis encephalopathy; aneurysm	27	43.9	MNI	8;4	PET	DOC < HC			

**Table 2:** Brain activity pattern in task-based map. Coordinates, extrema value, and corresponding Brodmann area, lobe, hemisphere, and regional labels are reported for each region resulting from the ALE map.

Region Number	Extrema Value Coordinates			Extrema Value	Brodmann Area	Hemisphere	Lobe	Label
	*x*	*y*	*z*					
1	−52	−30	10	0.013	41	L	Temporal	Superior Temporal Gyrus
1	−44	−30	10	0.010	41	L	Temporal	Superior Temporal Gyrus
2	46	−28	12	0.013	41	R	Temporal	Transverse Temporal Gyrus
2	50	20	10	0.010	41	R	Temporal	Transverse Temporal Gyrus

**Table 3:** Brain activity pattern in resting-state map. Coordinates, extrema value and corresponding Brodmann area, lobe, hemisphere and regional labels are reported for each region resulting from the ALE map.

Region Number	Extrema Value Coordinates			Extrema Value	Brodmann Area	Hemisphere	Lobe	Label
	x	y	z					
1	0	−36	32	0.034	31	L	Limbic	Cingulate Gyrus
1	2	−20	36	0.029	24	L	Limbic	Cingulate Gyrus
2	8	−16	6	0.031		R	Sub-lobar	Thalamus
2	−4	−14	6	0.024		L	Sub-lobar	Thalamus
3	4	12	24	0.018	24	R	Limbic	Cingulate Gyrus
3	4	8	42	0.013	24	R	Limbic	Cingulate Gyrus
4	14	14	8	0.026		R	Sub-lobar	Caudate
5	−32	6	54	0.020	6	L	Frontal	Middle Frontal Gyrus
6	−44	−70	40	0.021	39	L	Parietal	Angular Gyrus

**Table 4:** Activity patterns during rs-fMRI in MCS and VS patients. Coordinates, extrema value and corresponding Brodmann area, lobe, hemisphere, and regional labels are reported for each region resulting from the ALE map.

Region Number	Extrema Value Coordinates			Extrema Value	Brodmann Area	Hemisphere	Lobe	Label
	x	y	z					
MCS patients								
1	0	−36	32	0.022	31	L	Limbic	Cingulate Gyrus
2	4	−18	6	0.017	.	R	Sub-lobar	Thalamus
3	4	12	24	0.017	24	R	Limbic	Cingulate Gyrus
4	14	14	8	0.023	.	R	Sub-lobar	Caudate
5	−8	12	10	0.021	.	L	Sub-lobar	Caudate
VS patients								
1	10	−18	4	0.019	.	R	Sub-lobar	Thalamus
2	0	−38	34	0.015	31	L	Limbic	Cingulate Gyrus
2	4	−36	24	0.008	23	R	Limbic	Posterior Cingulate
3	4	−16	34	0.019	23	R	Limbic	Cingulate Gyrus
4	−6	−14	6	0.019	.	L	Sub-lobar	Thalamus

## Supplementary Materials

The following are available online at https://www.mdpi.com/2077-0383/9/3/828/s1, Figure S1: Biophysical head model, target map and multichannel tES optimized montages for stimulation of the motor cortex functional connectivity network.
