# Supplementary material for: Network Mapping of Connectivity Alterations in Disorder of Consciousness: Towards Targeted Neuromodulation
Source: J Clin Med. 2020 Mar 18;9(3):828. doi: 10.3390/jcm9030828 (PMC7141258; doi:10.3390/jcm9030828)
Supplement: Supplementary file 1 [file jcm-09-00828-s001.pdf]

Network Mapping of Connectivity Alterations in Disorder of Consciousness: Towards Targeted Neuromodulation

Lucia Mencarelli [1,2], Maria Chiara Biagi[3], Ricardo Salvador[3], Sara Romanella[1], Giulio Ruffini[3], Simone Rossi[1,4] and Emiliano Santarnecchi[1,2]

[1] Siena Brain Investigation and Neuromodulation Lab (Si-BIN Lab), Department of Medicine, Surgery and Neuroscience, Neurology and Clinical Neurophysiology Section, University of Siena, Italy
[2] Berenson-Allen Center for Non-Invasive Brain Stimulation, Beth Israel Deaconess Medical Center, Harvard Medical School, Boston, MA, USA
[3] Neuroelectrics, Cambridge, MA (US) and Barcelona (Spain)
[4] Human Physiology Section, Department of Medicine, Surgery and Neuroscience, University of Siena, Siena, Italy

Corresponding author:

Emiliano Santarnecchi

Berenson-Allen Center for Non-Invasive Brain Stimulation, Beth Israel Deaconess Medical Center,

Harvard Medical School, Boston, MA, USA

office +1-617-667-0326

mobile +1-617-516-9516

esantarn@bidmc.harvard.edu

Methods

Figure

Supplementary Figure S1

Realistic Biophysical Head Model

The realistic head model used in this study was built from a structural T1-weighted MRI of the single-subject template Colin27 (http://brainweb.bic.mni.mcgill.ca/brainweb) (Figure S1- a1). The 1 mm × 1mm × 1 mm resolution image was segmented into the main head tissues classes (Figure S1-a2) by using free software: the white-matter (WM) and grey-matter (GM) segmentations were obtained with FreeSurfer (https://surfer.nmr.mgh.harvard.edu), whereas cerebrospinal fluid (CSF), air filled sinuses, skull and scalp masks were obtained with MARS (Huang et al., 2013). The binary masks were combined into one 3D volume (Figure S1-a4) using custom Matlab scripts (r2018a, with Image Processing Toolbox and Iso2Mesh (Fang & Boas, 2009), which ensured that each tissue was surrounded by at least a 1 mm layer of another tissue and generated surfaces of all the tissues (Figure S1-a3). Sixty-four cylinders (1 cm radius 3 mm thickness) representing gelled PISTIM Ag/AgCl electrodes were placed in the scalp positions defined by the 10/10 EEG system, upon manual identification of the four fiducials (nasion, inion, left/right preauricular point) on the T1 image. The finite element volume mesh of the full head with electrodes (Figure S1-a5) was created using Iso2Mesh (Fang & Boas, 2009). The mesh comprised about 4.3 million tetrahedra. The mesh was then imported into Comsol (v5.3a) (http://www.comsol.com), where the tissues were assigned appropriate isotropic conductivities for the DC-low frequency range: 0.33, 0.008, 1.79, 0.40, 0.15 and 10^{-5} S/m respectively for the scalp, skull, CSF, GM, WM and air (Miranda, Mekonnen, Salvador, & Ruffini, 2013). The electrodes were represented as isotropic homogeneous conductive media with conductivity of 4.0 S/m. Laplace's equation was solved for the electrostatic potential (V) using Lagrange second order finite elements. As boundary condition, floating potential was imposed at the surface of the electrodes, to ensure a constant current injection.

The electric field (E-field) was obtained by taking the negative gradient of the potential. The E-field normal to the GM surface (E_n) was calculated for all the bipolar

montages having Cz as a common cathode (-1 mA) and each of the other electrodes as the anode (+1 mA). These results were used as the input to the optimization algorithm (Ruffini, Fox, Ripolles, Miranda, & Pascual-Leone, 2014), since the distribution of E_n for any montage involving these electrodes can be calculated as a linear combination of the bipolar unit-current E_n distributions multiplied by the electrode's current.

Montage Optimization

Montage optimizations (Figure S1-c) were performed using the Stimweaver algorithm, as described in Ruffini et al. (2014). Under the assumption of the lambda-E-model (Ruffini et al., 2013) for the interaction of the E-field with the neurons, the algorithm optimizes for the E_n component of the E-field. Positive/negative E_n values, directed towards/out-of the cortical surface, depolarize/hyperpolarize dendrites, soma and the axon terminal of pyramidal cells, thus modulating excitation.

The best montage is found by minimizing the least squares difference between the weighted target E_n-map (as defined in the next section) and the weighted E_n-field distribution induced by the montage, which defines the objective function. Minimization is constrained by safety limits for the currents (maximum current per electrode of 2mA, and maximum total injected current of 4mA), and by the maximum number of stimulation electrodes in the device (N = 8 here). The last condition is imposed by using a genetic algorithm that searches in electrode space for the constrained solution that better approximates the optimization objective function, as described in Ruffini et al. (2014).

Functional Connectivity Target Map

Functional connectivity volume images were converted into target networks for the optimization algorithm following a procedure already (Fischer et al., 2017) applied to the motor cortex network. These steps are briefly outlined here for completeness.

The FC correlation coefficient values r in the image (Figure S1-b1) are mapped onto the cortical surface of the reference brain (http://brainweb.bic.mni.mcgill.ca/brainweb), after registration to a common space (Figure S1-b2) (MNI).

Since we aim at promoting the activation (excitation) of the positively correlated areas and the inhibition of negatively correlated ones, regions with positive r were assigned positive target electric field E_n^{Target} = 0.25 V/m (excitatory), while regions with negative r were assigned to negative E_n^{Target} = −0.25 V/m (inhibitory).

The weighted target network for the optimization (Figure S1-b3) is created by applying different thresholds to ignore low and emphasize high correlation, and by linearly rescaling the r-values into absolute weights w (from 0 to 10). Note that, in order to reflect the correlation strength, the positive and negative r-values are saturated to different absolute maximum and minimum weights ($|w_{max}|$, $|w_{min}|$), with the higher between the two assigned to the regions with higher maximum absolute correlation $|r|$.

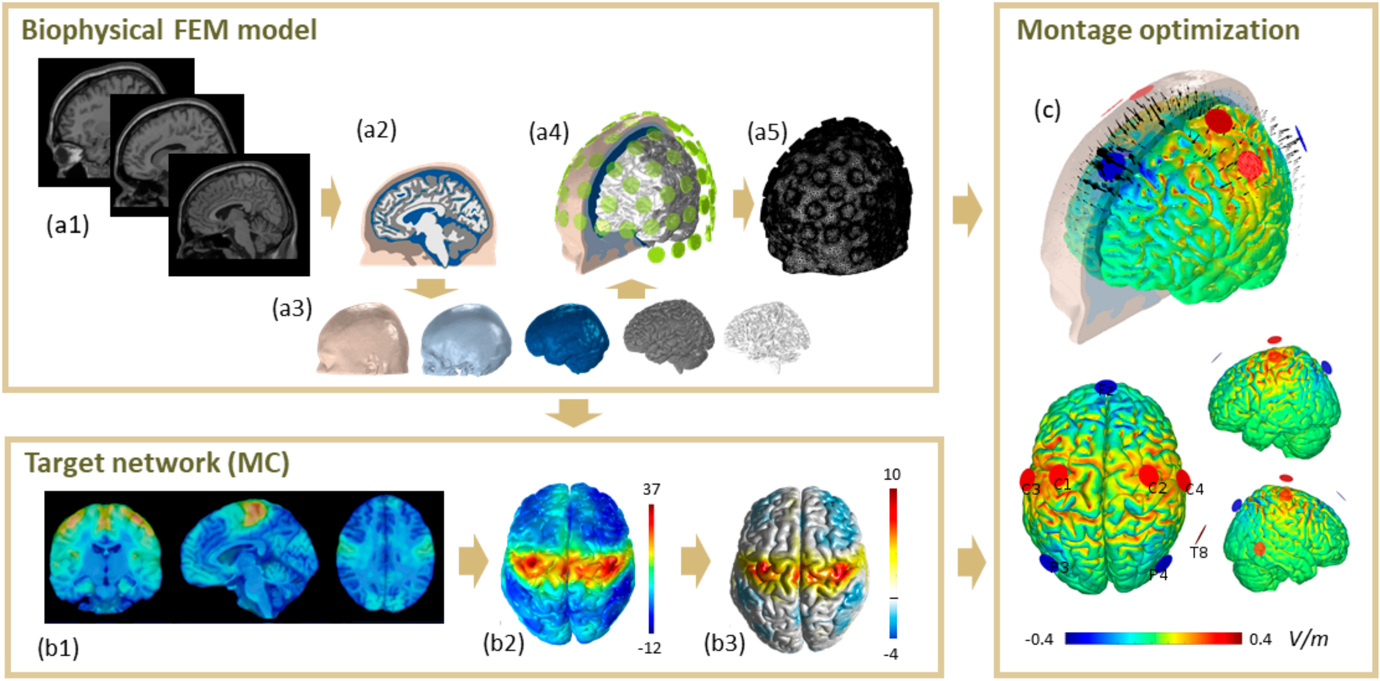

Figure 1. Biophysical head model, target map and multichannel tES optimized montages for stimulation of the motor cortex.

Biophysical Fem Model (a1–a5): the structural T1-weighted MRI (a1) of the template head (http://brainweb.bic.mni.mcgill.ca/brainweb) was segmented (a2) into the main tissue classes (scalp, skull, CSF and ventricles, grey matter, white matter); 3D surfaces of the tissues (a3) were created from the segmented volumes images and assembled into one head volume, where 64 1 cm-radius PISTIM

electrodes were placed on the scalp according the 10-10 EEG system (a4). The tissues were assigned conductivity in the DC regime (scalp = 0.33 S/m, skull = 0.008 S/m, CSF and ventricles = 1.79 S/m, GM = 0.4 S/M, WM = 0.13 S/m), and head model was meshed (a5) to calculate with finite elements the electric field induced on the cortex.

Target Map (b1–b3): the 3D image of the functional connectivity correlation (b1—in this example, the motor cortex network from Fisher et al., 2017) was remapped onto the cortical surface of the template brain (b2), and converted into a weighted target network (b3) by linearly rescale and saturation of the values (the values in the scale are multiplied by the sign of E_n^{Target} to display excitatory/positive and inhibitory/negative areas).

Montage Optimization (c1): normal electric field (V/m) induced on the cortex of the reference head model by the best multielectrode solution targeting the map in (b3), as determined by the optimization algorithm (Ruffini et al., 2014). For this example, the solution involves 7 electrodes, delivering a total maximum current 4mA. Anodes are shown in red, cathodes in blue; arrows represent current density.

References

Fischer, D. B., Fried, P. J., Ruffini, G., Ripolles, O., Salvador, R., Banus, J., … Fox, M. D. (2017). Multifocal tDCS targeting the resting state motor network increases cortical excitability beyond traditional tDCS targeting unilateral motor cortex. *NeuroImage*, *157*, 34–44. https://doi.org/10.1016/j.neuroimage.2017.05.060

Huang, Y., Dmochowski, J. P., Su, Y., Datta, A., Rorden, C., & Parra, L. C. (2013). Automated MRI segmentation for individualized modeling of current flow in the human head. *Journal of Neural Engineering*, *10*(6), 066004. https://doi.org/10.1088/1741-2560/10/6/066004

Miranda, P. C., Mekonnen, A., Salvador, R., & Ruffini, G. (2013). The electric field in the cortex during transcranial current stimulation. *NeuroImage*, *70*, 48–58. https://doi.org/10.1016/j.neuroimage.2012.12.034

Fang, Q., & Boas, D. A. (2009). Tetrahedral mesh generation from volumetric binary and grayscale images. *2009 IEEE International Symposium on Biomedical Imaging: From Nano to Macro*, 1142–1145. https://doi.org/10.1109/ISBI.2009.5193259

Ruffini, G., Fox, M. D., Ripolles, O., Miranda, P. C., & Pascual-Leone, A. (2014). Optimization of multifocal transcranial current stimulation for weighted cortical pattern targeting from realistic modeling of electric fields. *NeuroImage*, *89*, 216–225. https://doi.org/10.1016/j.neuroimage.2013.12.002

Ruffini, G., Wendling, F., Merlet, I., Molaee-Ardekani, B., Mekkonen, A., Salvador, R., Soria-Frisch, A., Grau, C., Dunne, S., Miranda, P., (2013). Transcranial

current brain stimulation (tCS): models and technologies. IEEE Trans. Neural. Syst. Rehabil. Eng. 21, 333–345
